# Pride Medical at Capitol Hill: A New Lesbian, Gay, Bisexual, Transgender, Queer/Questioning plus (LGBTQ+) Patient Care Option at Kaiser Permanente Mid-Atlantic States

**DOI:** 10.3390/healthcare11212816

**Published:** 2023-10-24

**Authors:** Mary Cabell Jonas, Keith Egan, Yi-Shin Sheu, Richard J. McCarthy, Michael A. Horberg

**Affiliations:** 1 Mid-Atlantic Permanente Research Institute, Rockville, MD 20852, USA; 2 Mid-Atlantic Permanente Medical Group, Rockville, MD 20852, USA

**Keywords:** LGBTQ+, patient care, inclusive care, primary care, OB/GYN

## Abstract

When accessing medical care, lesbian, gay, bisexual, transgender, queer/questioning plus (LGBTQ+) individuals face many known challenges, including stigma, discrimination, and health disparities. Transgender and nonbinary individuals often encounter physicians and staff who are not knowledgeable about gender-affirming services and the transition journey. Finding an affirming physician can be a trial-and-error process, causing concern and uncertainty. In 2021, Kaiser Permanente Mid-Atlantic States (KPMAS) researchers published a study examining the gaps in care and experience for transgender and nonbinary patients within the KPMAS healthcare system. KPMAS realized an opportunity to both close the gaps in care identified by transgender and nonbinary patients and enhance services for the broader LGBTQ+ patient community by creating Pride Medical at Capitol Hill—an additional and optional care site for individuals who identify as LGBTQ+. During the analysis timeframe of 30 June 2021 through 30 November 2022, 586 patients accessed care through 763 visits. A total of 675 visits (88%) were for primary care and 88 (12%) for OB/GYN. Over 50% (*n* = 384) of total visits were conducted virtually. The plurality of patients seen identified as a man (35%; *n* = 204) and gay (30%; *n* = 176). Postvisit survey results showed that 92% of survey respondents strongly agreed that the physician treated them with courtesy and respect, and 72% of survey respondents rated their overall care as excellent. Survey results show high acceptability of this program among the patients served. Pride Medical does not carve out care. The program offers patients access to a more specialized team of physicians—a similar model to other specialties—that is easily found by the division name Pride Medical. Layering additional specialty divisions on top of existing care, for interested patients, could be an option for other medical groups and health systems seeking to offer additional options of care for interested LGBTQ+ patients.

## 1. Background

Many LGBTQ+ patients find it difficult to connect with physicians who meet their unique needs [1,2,3]. Some individuals avoid medical care to protect themselves against hostility or discrimination [1,4]. One survey showed that 31% of LGBTQI+ individuals surveyed postponed or did not try to get needed medical care when sick or injured due to disrespect or discrimination from doctors or other healthcare providers [1]. Within KPMAS, feedback is obtained on an ongoing basis from various sources, including physicians, patient surveys, the clinical call center, research activities, and focus groups. Across these platforms, patients who are members of the LGBTQ+ community have provided feedback that encompassed many topical areas. Some specific pieces of feedback included: with over 1600 physicians in KPMAS, some patients found it challenging to know where to start gender-affirming services; some transmasculine patients expressed discomfort in the waiting rooms for OB/GYN services; and some LGBTQ+ patients expressed that there was no clear way to identify physicians with additional experience working with LGBTQ+ patients or find a primary care physician familiar with the healthcare needs of LGBTQ+ patients [5]. Executive leadership, physician champions, and the administrative project team considered this feedback, as well as research findings, to design an additional care option for LGBTQ+ patients named Pride Medical [6].

In the creation of Pride Medical, there was a mindful decision to add this program as a layer to existing primary care and specialty services. The leadership and the project team are very familiar with the LGBTQ+ community-health-center model, and this program was not designed to replicate that approach. The team did not want to carve out LGBTQ+ care and services, and the size of the program cannot accommodate all LGBTQ+ patients. LGBTQ+ patients can still access care through any physician. Rather, a goal was to make Pride Medical more visible and obvious to patients seeking a specialized level of care for certain needs.

Specialty care departments not only help patients find services but also gather more experienced physicians into a collaborative group. KPMAS applied a specialty care model to LGBTQ+ services within the health system by adding Pride Medical at Capitol Hill as an additional, optional, and specialized level of care for patients to access if they are interested or if their clinical needs require specialist expertise. It is important to reiterate that Pride Medical does not separate out LGBTQ+ care and services. Most LGBTQ+ patients still access care through primary care physicians and other specialists. Pride Medical is an additional layer of care above the existing primary care and specialty department care landscape. For some patients, having Pride Medical as its own division makes it easier to find the care they are seeking—whether it be with physicians experienced in providing gender-affirming care or with physicians having additional expertise or experience in meeting the health needs of the LGBTQ+ population group. For this reason, our organization feels this is a different model from a community clinic and is novel in having a structure more similar to a specialty department within a health system.

## 2. Organizational Context and Personal Content

Kaiser Permanente Mid-Atlantic States (Kaiser Foundation Health Plan and the Mid-Atlantic Permanente Medical Group) is an integrated care delivery system serving over 830,000 members in the District of Columbia, Maryland, and Virginia. The population of the Washington, DC metropolitan area has a very high percentage of LGBT individuals (9.8%) [7], and, although Kaiser Permanente is already a leader in offering equitable care to all communities [8,9], the leadership and the clinical teams felt that innovation could further improve and streamline the care options for LGBTQ+ patients. The leadership and clinical teams committed to establishing a new care option, named Pride Medical, that was easy to identify for LGBTQ+ patients seeking knowledgeable and experienced physicians.

Clinical and administrative leadership (Executive Medical Directors), physicians (infectious disease, OB/GYN, primary care), members of the research division, and individuals who identify as LGBTQ+ partnered together to develop Pride Medical. This program was launched in June 2021. During the analysis timeframe—30 June 2021 through 30 November 2022—Pride Medical included two board-certified adult and family medicine physicians and one board-certified OB/GYN physician with 10% of their clinical time devoted to the Pride Medical program; physicians work closely with one full-time nurse case manager. Physicians follow the World Professional Association of Transgender Health (WPATH) guidelines and are either WPATH members or in the process of certification. Primary care and OB/GYN care throughout KPMAS follows care standards, such as the United States Preventive Services Task Force (USPSTF), the American College of Obstetricians and Gynecologists (ACOG), and specialty-specific guidelines. Pride Medical includes adult gender-affirming care through the Gender Pathways program.

## 3. Problem

**Figure 1:**
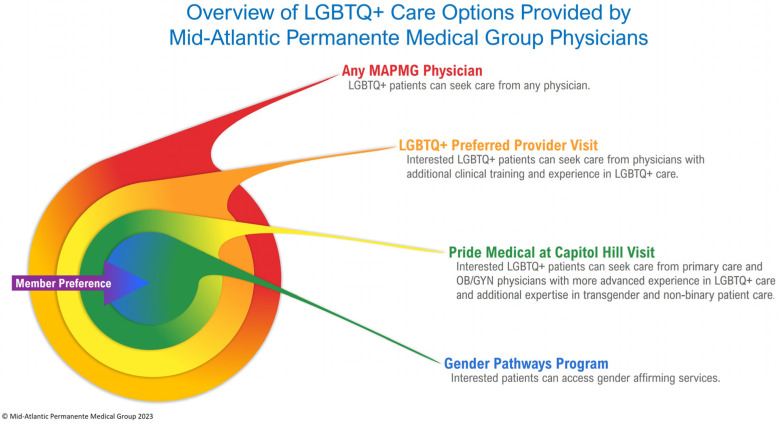
Overview of LGBTQ+ care options provided by Mid-Atlantic Permanente Medical Group physicians. Included within Pride Medical is the Gender Pathways Program, focused on gender-affirming care for transgender, nonbinary, and gender-diverse patients.

The goal was to create an easily accessible, additional care option for LGBTQ+ patients who were specifically looking for primary care and OB/GYN physicians with additional expertise in LGBTQ+ patient care who practiced virtually and in an inviting physical space (Figure 1). We sought to reach each LGBTQ+ population subgroup, particularly individuals who identify as transgender and who often experience broader gaps in care. We mindfully chose the Capitol Hill Medical Center location due to the high percentage of LGBTQ+ patients in the downtown Washington, DC area and its easy accessibility via public transit. The team sought to create a clinic space that clearly signaled acceptance and support of LGBTQ+ individuals, including signage and art. From a clinical care perspective, it was critical for the program to include primary care physicians, OB/GYN physicians, and case managers who were comfortable providing holistic, gender-affirming care to adult patients. The short-term program goals included understanding who sought care at Pride Medical, how patients wanted to access care, and how the program performed from a patient-experience perspective. Longer term program goals include improving performance on health metrics relevant to the LGBTQ+ community.

## 4. Solution

### 4.1. Pride Medical Program Development

Medical group leaders, infectious disease physicians, primary care physician leads with expertise in LGBTQ+ care, and members of the research division began designing Pride Medical in 2019. Over the course of one year, the team worked with administrative leads to structure the program and space, located at Capitol Hill Medical Center. The space offered an adjacently located room that was an ideal location for a new, more private waiting room for Pride Medical. The clinical leadership outlined the services to be provided (primary care and OB/GYN care), the visit structure (allowing patients who access most of their care from that physician (empaneled) and patients who access most of their care from a different physician (nonempaneled) to access visits), schedules, patient experience surveys and goals, and the initial quality-improvement metrics for the program.

Patients can see Pride Medical physicians for individual visits or join the patient primary care panel or OB/GYN panel of a Pride Medical physician. Joining a panel means the doctor becomes the patient’s primary doctor. Accessing individual Pride Medical visits means that the patient primarily sees a non-Pride Medical primary care doctor, but occasionally accesses care through Pride Medical doctors. The ability for patients to access certain care from Pride Medical is similar to a specialty department.

Visits can be booked through the patient portal or call center. The current model is 10% time per physician, during the initial development phase of the program. Over time, the physicians will increase the time dedicated to Pride Medical. All visit lengths are 20 min, with the exception of the OB/GYN procedural visit, which is 40 min. The visits are either in person or virtual via a secure platform. Pride Medical has branded wayfinding signage and a dedicated waiting room with artwork by LGBTQ+ artists. All KPMAS staff and physicians are trained in LGBTQ+ cultural sensitivity and patient service using an internally developed training module that includes definitions of terms, learning modules, and quizzes that must be passed before Continuing Medical Education (CME) or Continuing Education Units (CEU) credit is granted (information available upon request). Pride Medical staff are also expected to view additional tutorials about LGBTQ+ patient care and engage with physician leads to discuss topics that arise during care delivery. We sent a postvisit survey to all patients who complete an in-clinic or virtual Pride Medical visit; patients could receive the survey for both in-clinic and virtual visits twice per calendar year, and we sent the survey through the patient portal within 24 h of the visit. Administrative leads developed the postvisit survey in collaboration with individuals who personally identify as members of the LGBTQ+ community; the survey team specifically included questions that transgender individuals felt would be important to monitor patient experience. Key questions included: please rate your agreement with how the staff treated you with courtesy and respect (five-point scale, Strongly Disagree to Strongly Agree; rating is for Physician) and how would you rate the overall care you received at your visit today (five-point scale, poor to excellent). If a patient self-identified as transgender or nonbinary on the survey, they also received the following questions: please rate the transgender or nonbinary knowledge and care provided by the staff that you saw today (five-point scale, poor to excellent; rating is for physician) and please rate the trans friendliness of the staff that you saw today, including the use of your preferred name and pronouns (five-point scale, poor to excellent; rating is for physician). We reported the cumulative results for the questions shown, for the time period of study. Respondents could skip any question and write free text comments.

### 4.2. Initial Results

The KPMAS Institutional Review Board (IRB) reviewed and approved the project. The IRB approved a waiver of informed consent for this study. The program team examined in-person and virtual visits for Pride Medical primary care and OB/GYN services for adult patients aged 18 years or older, between 30 June 2021 through 30 November 2022. During the time frame studied, 586 unique patients received care at Pride Medical within a total of 763 visits. Most patients were White (*n* = 273; 47%), followed by Black (*n* = 194; 33%), Hispanic/Latino (*n* = 62; 11%), Asian (*n* = 25; 4%), Other (*n* = 15; 2.5%), Unknown (*n* = 8; 1.4%), American Indian/Alaskan Native (*n* = 6; 1%), Decline to State (*n* = 2; 0.3%), and Native Hawaiian/Pacific Islander (*n* = 1; 0.17%); Table 1). Nearly half of the patients seen were between the ages of 26-35 (*n* = 273; 46.6%); the next highest group was aged 18–25 (*n* = 145; 24.7%). The primary language spoken by patients was English (*n* = 562; 95.9%). Most patients had a median household income in the range of $100,001–150,000 (*n* = 147; 25.1%) followed by $50,001–75,000 (*n* = 132; 22.5%). We based the median household income for each patient on the tract income level derived from patient geographic areas, as specified in Federal Information Processing System (FIPS) codes.

Gender identity and sexual orientation data were collected using the Sexual Orientation and Gender Identity (SOGI) electronic health record (EHR) questionnaire, which is a feature of the EHR in which individual patient sexual orientation and gender identity information are documented. Patients could choose to decline to answer the questions. Pride Medical patients most often identified as a man (*n* = 204; 34.8%), followed by transgender (*n* = 126; 21.5%) and nonbinary (*n* = 85; 14.5). The majority of patients identified their sexual orientation as gay (*n* = 176; 30%), followed by a sexual orientation of something else (*n* = 96; 16.4%); 9% (*n* = 53) of patients were living with HIV.

**Figure 2:**
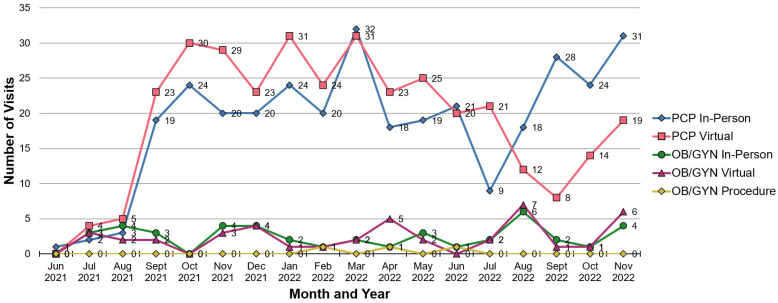
Pride Medical visits by type and month. PCP, Primary Care Physician; OB/GYN, Obstetrics and Gynecology.

Visits included in-person primary care, virtual primary care, in-person OB/GYN (including medical and procedural visits), and virtual OB/GYN visit types. A total of 675 visits (88%) were for primary care and 88 (12%) for OB/GYN care (Figure 2). Of the total visits that were conducted, 50% (*n* = 384) were virtual (video or phone); 51% (*n* = 342) of primary care and 47% (*n* = 42) of OB/GYN visits were conducted virtually. Nearly all visits (99%; *n* = 760) were 20 min in length; less than 1% (*n* = 3) of total visits were OB/GYN Procedural visits (in-person 40 min visit). We excluded canceled visits, no-show visits, and physician–patient secure messages.

Over 60% (*n* = 355) of patients accessing primary care and 96% (*n* = 561) accessing OB/GYN care were not part of a Pride Medical physician’s patient panel; this means the patient typically accesses primary and OB/GYN care from a non-Pride Medical physician in another location. Most patients seeking care at Pride Medical were part of the District of Columbia/Suburban Maryland Kaiser Permanente service area (*n* = 426; 72.7%), although all service areas across the region were represented [Northern Virginia *n* = 100 (17.1%) and Baltimore *n* = 60 (10.2%)]. We designated the service area based on the patient’s primary location, derived from service utilization.

**Figure 3:**
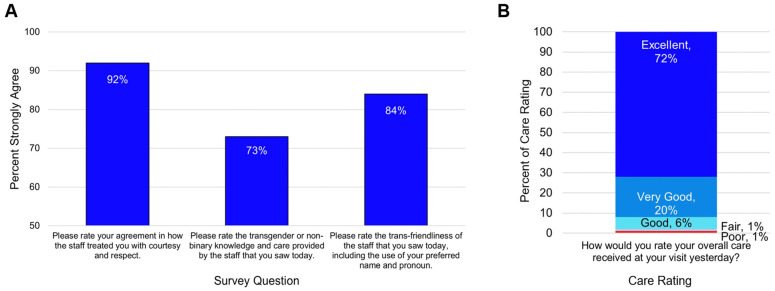
Quantitative survey feedback. (**A**) Percentage of responses “Strongly Agree” with the indicated survey question, for staff member physician. (**B**) Percentages of each care rating for the indicated survey question. Combined survey responses from both in-person and virtual visits are shown.

Patients received an optional postvisit survey developed in collaboration with physicians, project administrators, and members of the LGBTQ+ community. Within the timeframe studied, 108 respondents completed the survey (Figure 3). For the question, “Please rate your agreement in how the staff treated you with courtesy and respect” 92% (*n* = 105) of survey respondents answered Strongly Agree for the physician staff member. For the question, “How would you rate the overall care you received at your visit today?” 72% (*n* = 78) of survey respondents answered excellent.

Patients who self-identified on the survey as transgender or nonbinary received the question, “Please rate the transgender or non-binary knowledge and care provided by the staff that you saw today”, and 73% (*n* = 63) of survey respondents answered excellent for the physician staff member. For the question “Please rate the trans-friendliness of the staff that you saw today, including the use of your preferred name and pronouns” 84% (*n* = 63) of respondents answered excellent for the physician staff member.

## 5. Unresolved Questions and Lessons for the Field

Although the initial results show success, the implementation of Pride Medical did face challenges, some that were resolved and some that require future improvement.

### 5.1. Technology Issues

According to the Pride Medical survey results, most respondents gave the highest rating to describe the experiences felt. The respondents viewed the physicians as knowledgeable, respectful/courteous, and trans friendly—which indicates the team is meeting the goal of creating a safe and affirming care option for gender-diverse patients. For the trans friendliness survey question, the team observed a difference in scores between in-person and virtual visits, with virtual visits scoring lower. In exploring the discrepancy, the team uncovered that the video-visit IT platform was showing the patient’s legal name rather than their chosen name, which resulted in complaints about trans friendliness which then extended into the medical visit. The team has addressed this technical issue to ensure that the preferred name now displays for patients within the video-visit platform (preferred name can include legal name, name, or nickname and is documented and shown for all patients regardless of gender identity).

### 5.2. Underuse by Subpopulations

A fairly low percentage of Pride Medical patients identify as lesbian (8.7%). Lesbian patients may already feel comfortable seeking care from their usual OB/GYN, rather than seeking out the Pride Medical OB/GYN for care. Additional research is warranted to understand how lesbian patients select physicians and how Pride Medical can better serve this population.

### 5.3. Missing Data

Although Pride Medical physicians strive to complete the SOGI EHR questionnaire for all Pride Medical patients, data on sexual orientation and gender identity was unavailable for 16.2% of patients seen—indicating an area for focus and improvement. Clinic process metrics now include a goal to collect SOGI data on all Pride Medical patients and this data is tracked on an ongoing basis. The SOGI EHR questionnaire includes options for patients who choose not to disclose their sexual orientation or gender identity.

### 5.4. Virtual Care Delivery

The time period of the study was during the COVID-19 public-health emergency timeframe. This may have increased the percentage of visits accessed virtually. However, even upon publication, visit distribution remains approximately 50% virtual. Because Kaiser Permanente operates under a membership-based, prepaid payment model, telemedicine services existed pre-COVID-19 pandemic and will remain the same after telemedicine regulations change. Other healthcare providers seeking to replicate this model should consider the challenges they may face with telemedicine reimbursement.

## 6. Conclusions

The Kaiser Permanente Mid-Atlantic States Pride Medical at Capitol Hill program is serving a diversity of LGBTQ+ patients, through both in-person and virtual access channels. The survey results show high acceptability of this program among the patients served. The conclusions outlined below provide important lessons for developing similar care options by other healthcare systems nationwide.

### 6.1. Offer Virtual and In-Person Care Options

Virtual visits were critical. Nearly half of all primary care and OB/GYN visits were conducted virtually, indicating that even proximal patients prefer to take advantage of virtual visits when possible. This result underscores the importance of creating a program with virtual visits that can reach near or distant patients.

### 6.2. Offer a Model to Be Accessed as Specialty Care

The majority of patients seen were not on the Pride physician’s panel, meaning the patient typically accesses most of their medical care from a different primary care and/or OB/GYN physician. This means that a proportion of patients are seeking out the Pride Medical program for specific clinical needs and not necessarily as the “medical home” for the patients’ general healthcare. Offering a program structure that enables patients to seek care with physicians in more of a specialist model, for specific needs, is clearly appealing to patients.

### 6.3. Adapt to Reach all Groups and Grow with the Need

One key goal of the Pride Medical program was to offer a more specialized group of physicians for transgender and nonbinary patients to access gender-affirming care. The results show that 21.5% of the Pride Medical patients included in this study timeframe are transgender (*n* = 126; transgender man 11.4% and transgender woman 10.1%). Of the 8.5% of patients who identified as straight, many are transgender, but a percentage are known to be the straight partners of a transgender patient. These partners come to Pride Medical seeking support and information about their transgender partner’s transition process (personal communication). However, Pride Medical should continue to adapt to reach all LGBTQ+ population subgroups. The patients seen at Pride Medical to date tend to be younger—with 71.3% under age 35—and identify as a man and gay. As a result of the rising demand for Pride Medical services, the program has expanded to include more physicians and more time per physician devoted to Pride Medical visits. As of 2023, the Pride Medical team now consists of three primary care physicians and two OB/GYN physicians. Future directions for this program include conducting an analysis of the LGBTQ+ patient subgroups accessing each Pride Medical visit type, and the clinical reasons cited for the visits. In order to make more patients aware of the Pride Medical program, the project team will continue to share information with Kaiser Permanente members as well as news outlets that serve the LGBTQ+ community.

### 6.4. The Program Should Be Additive and Specialized

LGBTQ+ community clinics have a long history. Pride Medical is not intended to replicate this model and does not intend to attract all LGBTQ+ patients into the program. Most LGBTQ+ patients will remain with their existing care providers who are meeting their needs. Rather, Pride Medical is an additional, specialized care option for patients with specific clinical needs or preferences for care. The program is layered on a robust foundation of physicians working to provide high-quality care to all patients and who are trained on the care and service needs of all groups. The program is meant to operate more as a specialty model, particularly for transgender patients seeking gender-affirming services. Knowing that Pride Medical will only serve a subset of the total LGBTQ+ population, future research will explore why patients seek care through Pride Medical and explore other factors that may have influenced their interest in this program (e.g., experiences of discrimination in general or in the medical environment or have specialized clinical needs).

Our results indicate that Pride Medical shows promise in offering an additional, optional site of specialty care for patients who are part of the LGBTQ+ community. Unlike carved-out models of LGBTQ+ care, Pride Medical offers additional service options that are additive to a patient’s regular primary care and OB/GYN care. Pride Medical may serve as a model for other healthcare providers aiming to offer additional, specialized LGBTQ+-centered patient care options.

## Figures and Tables

**Table 1:** Sociodemographic characteristics of patients with Pride Medical visits.

	Full Sample (*n* = 586)	
	*n*	%
Race		
White	273	46.6
Black/African American	194	33.1
Hispanic/Latino	62	10.6
Asian	25	4.3
Other	15	2.6
Unknown	8	1.4
American Indian/Alaska Native	6	1.0
Decline to state	2	0.3
Native Hawaiian/Other Pacific Islander	1	0.2
Age		
18–25	145	24.7
26–35	273	46.6
36–45	100	17.1
46–55	47	8.0
56–65	14	2.4
66+	7	1.2
Language spoken		
English	562	95.9
Spanish	11	1.9
Other ^a^	6	1.0
Not available	5	0.9
Sign Language	2	0.3
Home service area		
DC-SM	426	72.7
NOVA	100	17.1
Baltimore	60	10.2
Gender identity		
Man	204	34.8
Nonbinary	85	14.5
Not Available	80	13.7
Transgender Man	67	11.4
Transgender Woman	59	10.1
Woman	54	9.2
Other	23	3.9
Genderfluid	7	1.2
Questioning	4	0.7
Choose not to disclose	3	0.5
Legal sex		
Male	351	59.9
Female	235	40.1
Sexual orientation		
Gay	176	30.0
Something else	96	16.4
Not Available	95	16.2
Bisexual	74	12.6
Lesbian	51	8.7
Straight (not lesbian or gay)	50	8.5
Choose not to disclose	24	4.1
Do not know	20	3.4
HIV status		
Negative	533	91.0
Positive	53	9.0
Empaneled with Pride PCP		
No	355	60.6
Yes	231	39.4
Empaneled with Pride OB/GYN		
No	561	95.7
Yes	25	4.3
Median household income		
$15,000–$30,000	7	1.2
$30,001–$50,000	42	7.2
$50,001–$75,000	132	22.5
$75,001–$100,000	122	20.8
$100,001–$150,000	147	25.1
$150,001–$200,000	41	7.0
>$200,000	7	1.2
Not Available	88	15.0

^a^ Includes Vietnamese, Tigrinya, Latin, French, and Miscellaneous. DC-SM, District of Columbia and Suburban Maryland; NOVA, Northern Virginia.

## Data Availability

The data presented in this study are available upon request from the corresponding author. The data are not publicly available due to patient privacy.
